# Potential cost-effectiveness and benefit-cost ratios of adult pneumococcal vaccination in Germany

**DOI:** 10.1186/2191-1991-2-4

**Published:** 2012-03-30

**Authors:** Alexander Kuhlmann, Ulrike Theidel, Mathias W Pletz, J-Matthias Graf von der Schulenburg

**Affiliations:** 1 Centre for Health Economics, Königsworther Platz 1, Hannover, 30167, Germany; 2 herescon gmbh, Hannover, Germany; 3 Division of Gastroenterology, Hepatology and Infectious Diseases, Jena University Hospital, Jena, Alemanya

**Keywords:** Cost, Effectiveness, Pneumococcal polysaccharide vaccine, Pneumococcal conjugate vaccine, Adult, Benefit-cost

## Abstract

**Background:**

Invasive (IPD, defined as detection of pneumococci in sterile body fluids like meningitis or bacteremic pneumonia) and non-invasive *Streptococcus pneumoniae* infections (i.e. non-bacteremic pneumonia, otitis media) in adults are associated with substantial morbidity, mortality and costs. In Germany, Pneumococcal polysaccharide vaccination (PPV23) is recommended for all persons >60 years and for defined risk groups (age 5–59). The aim of this model was to estimate the potential cost-effectiveness and benefit-cost ratios of the adult vaccination program (18 years and older), considering the launch of the pneumococcal conjugate vaccine for adults (PCV13).

**Methods:**

A cross-sectional steady state Markov model was developed to estimate the outcomes of PCV13, PPV23 vaccination schemes and ‘no vaccination’. Conservative assumptions were made if no data were available for PCV13 and PPV23 respectively. The effectiveness of individual pneumococcal vaccination in adults was adjusted for expected indirect effects due to the vaccination in infants. Data on incidences, effectiveness and costs were derived from scientific literature and publicly available databases. All resources used are indicated. Benefit-cost ratios and cost-effectiveness were evaluated from the perspective of the German Statutory Health Insurance as well as from social perspective.

**Results:**

Under the assumption that PCV13 has a comparable effectiveness to PCV7, a vaccination program with PCV13 revealed the potential to avoid a greater number of yearly cases and deaths in IPD and pneumonia in Germany compared to PPV23. For PCV13, the costs were shown to be overcompensated by monetary savings resulting from reduction in the use of health care services. These results would render the switch from PPV23 to PCV13 as a dominant strategy compared to PPV23 and ‘no vaccination’. Given the correctness of the underlying assumptions every Euro spent on the PCV13 vaccination scheme yields savings of 2.09 € (social perspective: 2.16 €) compared to PPV23 and 1.27 € (social perspective: 1.32 €) compared to ‘no vaccination’, respectively.

**Conclusions:**

Results of the model indicate that the health economic benefit of immunizing adults with PCV13 can be expected to outperform the sole use of PPV23, if the effectiveness of PCV13 is comparable to the effectiveness of PCV7.

## Background

*Streptococcus pneumoniae* (pneumococcus) is worldwide a leading cause of infections associated with high case fatality rates like sepsis, meningitis and pneumonia. Of the 91 identified pneumococcal serotypes, 10 to 15 pose major risks to morbidity and mortality, particularly in young children (age ≤5 years (y)), elderly (age ≥65 y) and immunocompromised patients (all age groups). In addition, pneumococcus is the most common cause of community-acquired pneumonia (CAP) in adults.

In 2001, a 7-valent pneumococcal conjugate vaccine (PCV7) became available in Europe for children. Higher-valent PCVs were launched in 2009, a 10-valent (PCV-10) and a 13-valent pneumococcal conjugate vaccine (PCV13). Since August 2006 the Standing Vaccination Committee at the German Robert-Koch Institute (STIKO) recommends vaccination of all children less than two years of age. In addition, children at risk should be vaccinated between the ages of 3 and 5 years, since August 2010 with the 13-valent pneumococcal conjugate vaccine. The conjugate vaccine induces a strong antibody response in children and reduced significantly the incidence of IPD in Germany [1] and other European countries.

Contrary to the recommendations for children, the 23-valent pneumococcal polysaccharide vaccine (PPV23) is still the recommended vaccine in Germany for prevention of pneumococcal diseases in elderly (age ≥60 y) and adults at risk despite the ongoing controversy regarding its effectiveness. A recently published meta-analysis and a series of published studies did not confirm the effectiveness of PPV23 and concluded that policy-makers should reconsider their current recommendations for the use of the pneumococcal polysaccharide vaccine, especially when pneumococcal conjugate vaccine get licensed for adults [2-12].

The T-cell dependent immune response of PCV13 induces antibody titres in elderly comparable to those induced in infants. [13] Vaccination studies have shown that antibody levels after PCV13 were at least similar or superior for most vaccine shared serotypes to that induced by PPV23. However, implementing a PCV13 vaccination scheme in adults will cause additional costs in the German health care system due to the higher price of the vaccine. Therefore, this analysis aims to evaluate the benefit-cost ratio and cost-effectiveness of an adult vaccination program in individuals older than 50 years compared to the existing PPV23 recommendation for adults as well as to ‘no vaccination’.

## Methods

### Model structure

To compare the benefit-cost ratio and cost-effectiveness of three different pneumococcal vaccination strategies (PPV23, PCV13 and ‘no vaccination’) in adults, we updated and extended a recently developed Markov model. The analysis focused on a comparison between a PCV13 and a PPV23 vaccination strategy. Therefore, we simulated a setting in which both vaccination programs were fully established according to the vaccination recommendations in Germany. For the following, we define this setting, in which all new entrants of the target groups were being vaccinated or re-vaccinated and all other individuals were already immunized according to the vaccination recommendations and assumed vaccination rates, as a steady state. Hence, time series of starting and establishing a vaccination were not included in the analysis. The evaluation took the perspective of the German Statutory Health Insurance (SHI), and additionally estimated social work loss costs. Outcomes and costs of each vaccination strategy for one year in a steady state were calculated in two steps.

Due to a lack of epidemiological data, we estimated the population at risk (distinguishing between normal, moderate and high risk of pneumococcal disease) in 83 age groups (18–100 years of age) in the above mentioned setting as well as the proportion of immunized individuals due to vaccination in each age/risk group using a Markov state transition model with a time horizon of 100 years. The cycle length was one year. In particular, the model took account for effects of immunized individuals transiting to other risk groups over time. The population simulation started with healthy and unvaccinated newborns developing age-specific risk of comorbidities with increasing age which are associated with moderate or high risk of pneumococcal disease. Each risk group got vaccinated according to the strategies described below. Group members at moderate or high risk remained in their risk group during the simulation. Transition to death was possible from all states. In order to account for the age distribution of the German population, each age group was weighted according to the German population structure in the year 2008 [14].

Secondly, for the modelling of cost-effectiveness number of cases and deaths per year due to pneumococcal diseases were estimated for each vaccination strategy based on the results of the simulated population. To avoid interferences with the recently published children model, children and adolescents were excluded. Risk and age specific morbidity and mortality rates as well as the effectiveness of the vaccination were taken into account. In addition, the risk model calculated the yearly costs of the vaccination programs and yearly treatment costs for pneumococcal diseases.

The time horizon of one year avoided discounting, a topic of high relevance and unclear positioning in the indication of prevention. On the other hand, the cross sectional design of the model neglected costs associated with the implementation of a new vaccine combined with sunk costs of a new catch-up vaccination, which might be of interest regarding the budget impact of a new vaccination program.

The model was constructed in Microsoft Excel 2007.

### Vaccination strategies/choice of comparators

In Germany PPV23 vaccination is generally recommended for adults ≥60 y and for all patients >5 y with comorbidities and increased risk of pneumococcal disease. Since 2009, revaccination every 5 years is restricted to patients with immuno-compromising conditions due to frequent adverse events at the site of injection and questionable effectiveness of the vaccine [15].

To estimate the effects of PCV13 vaccination, a hypothetical vaccination program with vaccinating adults older than 50 years (branch 1 = PCV13) was compared to the existing PPV23 recommendation (branch 2 = PPV23). In the PCV13 branch, we assumed that adults at risk were vaccinated with PPV23 until they reached the age of 50. Thereafter they switched to PCV13 when they were revaccinated. That goes in line with current recommendation for pneumococcal vaccination and the targeted label for PCV13. Adults developing comorbidities associated with moderate or high risk of pneumococcal disease after the age of 50 were initially and revaccinated with PCV13 as were seniors with normal risk who got their initial vaccination at the age of 60, analogous to the PPV23 recommendation. For PCV13 the need for a revaccination in adults has not been established. Nevertheless we assumed a decennial booster, based on the experience of pneumococcal conjugate vaccination in children [16-18].

Due to the lack of data, we had to assume vaccination rates for initial and booster vaccination. Considering that the population at risk gets a higher awareness, initial vaccination rates were assumed to be higher than in the risk free population (40% vs. 25%). PPV23 booster vaccination was only considered for every patient in the high risk groups according to STIKO recommendations [15,19]. In contrast, PCV13 booster vaccination was considered for every patient at risk and every second without any risk. Due to the assumed superior effectiveness of PCV13 restrictions regarding revaccination are not expected.

In a third branch we modelled the ‘no vaccination’ scenario representing the strategy not to prevent but to treat pneumococcal disease. We included this scenario to analyse the total effects of both vaccines in addition to the incremental effects of PCV13 versus PPV23.

### Epidemiology

**Figure 1:**
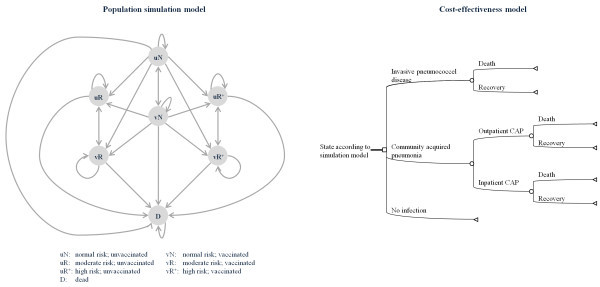
Simplified structure of the model.

The simulated model population (see Figure 1, population simulation model) size was around 82,000,000 individuals representing the estimated German population of adults in 2008. [14] The age structure of the initial cohort and the number of patients at risk, estimated according to data of Fleming et al., are shown in Table 1. Calculation of life expectancy was based on the German life tables from 2007/2009 [20], while the population structure was taken from the German Federal Statistical Office (2008) [14]. In 2008, the overall German population of 82.0 million continued to decrease. Following the trend of recent years the age group >60 y continues to grow. To model the cost-effectiveness, a population size of 69,200,000 Mio was used (age 18–100), considering different risk groups.

**Table 1:** Simulated population size (total population)

**Age group**	**General population**	High risk*	Moderate risk*
< 5	2,730,000	4,600	16,000
5 - 17	10,070,000	75,100	260,000
18 - 39	21,700,000	405,000	1,400,000
40 - 59	25,500,000	1,001,000	2,777,000
60+	22,000,000	2,874,000	7,415,000
Total	82,000,000	4,359,700	11,868,000

*Estimated according to Fleming et al. [21].

In contrast to the health economic endpoint, the epidemiological endpoints are avoidable episodes of IPD and non-bacteremic CAP in an inpatient setting as well as in an outpatient setting. Historical incidences for IPD in adults are taken from a regional surveillance study in North-Rhine Westphalia prior to the general PCV vaccination recommendation for children. The age-specific IPD incidence was adjusted for underreporting with a factor of 2.7, due to the very low standard with regard to the collection of blood cultures in Germany.

The burden of pneumococcal pneumonia is high and the most serious complication is a bacteremic course associated with a high case fatality rate. The CAP incidence incorporated all causes as detailed microbiological diagnosis is not performed on a regular basis. Data on incidence of CAP treated in hospital (“inpatient CAP”) are based on the German hospital admission data (2008). [22] The BQS-Report documented that 200,000 CAP episodes were treated within hospital every year, which is very close to the numbers incorporated in our model based on admission data. [23] Age specific incidence of CAP treated in primary care settings (“outpatient CAP”) were derived from a representative pharmaceutical prescription panel which results (12 episodes in 1,000 adults) were similar to results reported in the literature. [24] For Schleswig-Holstein (a Northern region in Germany), incidence of CAP ranged between 3.7 and 12.3 cases per 1,000 adults.

Due to the lack of valid data, odds ratios for IPD and CAP incidences in moderate and high risk population were assumed to be 1.5.

The IPD case fatality rate (CFR) for adults was taken from the literature (US 2000–2004). In comparison, the German CFR due to streptococcal and other sepsis cases (ICD A40, A41, A49.1 and G00.1) in the age groups <15, 15 - < 45, 45 - < 65, 65 - < 75, 75 - < 85 and ≥85 was 1.20%, 3.56%, 7.23%, 9.63%, 14.93%, 21.24%. [22] The study of Dzupova et al. confirmed a CFR for adult pneumococcal meningitis of 20%.

The 6 months CFR of inpatient CAP was based on data from the German CAPNetz [25]. While the CFR in CAP in outpatient settings amounted to about 1%, CFR in inpatient CAP was higher (see Table 2). CFR for outpatient CAP was assumed to be 0% for patients <60 y and 0.5% for patients being 60y and older.

**Table 2:** Incidence (per 100,000 individuals) and Case Fatality Rate (CFR)

**Age group**	**IPD**		**Inpatient CAP**		**Outpatient CAP**	
	**Incidence**	**CFR**	**Incidence[**[22]**]**	**CFR[**[25]**]**	**Incidence[**[24]**]**	**CFR#**
18 - 39	0.5	14.9% ^#^	48.9	1.6%	574.7	0.0%
40 - 49	6.8	14.9% ^#^	80.4	4.3%	777.3	0.0%
50 - 59	13.3	14.9% ^#^	149.7	6.3%	1,074.0	0.5%
60 - 69	23.0	14.9% ^#^	350.4	10.8%	1,459.3	0.5%
70 - 79	41.6	16.5%	768.7	16.8%	1,465.8	0.5%
80+	53.7	27.4%	1,897.8	22.6%	2,025.2	0.5%

^§^ including a factor of 2.7 for underreporting (29), ^#^ assumption,

CAP: community acquired pneumonia, CFR: case fatality rate, IPD: Invasive Pneumococcal Disease.

### Effectiveness

The effectiveness of different pneumococcal vaccines depends on immunization level, vaccine efficacy in invasive and non-invasive pneumococcal diseases differentiated to age and risk groups, serotype coverage, vaccine effectiveness against cross-reactive serotype, and duration of protection, replacement, revaccination, indirect (herd) effects in the same target group, and indirect (herd) effects from vaccination of children. Data for vaccine efficacy and effectiveness are shown in Table 3.

**Table 3:** Efficacy and effectiveness data for pneumococcal vaccines

	**PCV13**		**PPV23**	
	**IPD**			
**Age Group**	Efficacy	Serotype Coverage	Efficacy	Serotype Coverage
**18 - 39**	93.9% [26]^*^	67.9%	74.0%^*^[9]	83.3%
**40 - 49**		75.4%		78.5%
**50 - 59**		63.4%		78.9%
**60 - 69**		63.9%		83.6%
**70 - 79**		71.4%		78.3%
**80 - 89**		74.8%		84.5%
**≥ 90**		76.2%		90.5%
				
	**Inpatient CAP / Outpatient CAP (all cause)**			
**Age Group**	Effectiveness		Effectiveness	
**All**	26.0%/6% [27,28]		0%/0% [6,9,19]	

* = multiplication.

According to published data and adaption of general recommendation, we calculated IPD effectiveness of both vaccines using efficacy data, adjusted to serotype distribution in Germany for all age groups.

For PPV23, IPD data were derived from a Cochrane meta-analysis [9] reporting an correlate IPD efficacy of 74% (with no present statistical heterogeneity when all RCT´s were considered). However, the reported correlate IPD efficacy was not representative for subgroups e.g. population with chronic illness in high income countries. With a 95% CI efficacy ranges overall from 56% to 85%. So we used the upper and lower bound for sensitivity analysis. In contrast, the reported efficacy data for CAP were inconclusive with substantial statistical heterogeneity [9]. Incorporating the findings from a Canadian meta-analysis [6] the conclusion can be drawn, that PPV23 does not appear to be effective in preventing CAP. Therefore, we assumed in the base case analysis that PPV23 is ineffective in preventing CAP. We abandoned this assumption in the sensitivity analysis by calculating the economical outcomes of PPV23 effectiveness against inpatient CAP according to Maruyama et al. [29].

For PCV13, assumption on IPD efficacy was based on clinical data for PCV7 in children for all patients, expecting similar levels of efficacy against the additional 6 serotypes which are not included in PCV7. Serotype coverage was calculated on the basis of data reported by Reinert et al. [30] Effectiveness of initial vaccination on inpatient and outpatient CAP for PCV13 was assumed to be 26% and 6%, respectively, according to effectiveness data on PCV7 [27,28].

We assumed that vaccines were equally effective against all vaccine serotypes, and further assumed that effectiveness of revaccination with PCV13 and PPV23 was the same as that of initial vaccination. Immunization due to vaccination with PCV13 and PPV23 was assumed to be effective for 10 years and 5 years, respectively. Then the effects wane completely.

In our model, we assumed that childhood vaccination programs with PCV7 were already established. Since children vaccinated with a conjugated vaccine are unlikely to be carriers for the seven vaccine serotypes, they can no longer transmit these to others. In particular, elderly benefit from this indirect protection. Vaccinated children, vaccinated successfully, are no carrier of pathogens. Indirect effects reduced probability of infection in the elderly. Evidence for indirect effects was reported from the US and Australia [31-36].

Markov cohort models are not able to directly capture indirect herd effects based on transmission dynamics of infectious disease. Hence, we included one additional parameter “herd effects” in our model. To address indirect effects from the childhood vaccination, the effectiveness of adult vaccination was reduced by a factor of “herd effects”. The disease and age group specific values for this correction factor are shown in Table 4. The figures were calculated based on US data from Ray et al. [34] and Pilishvili et al. [37] adjusted for German serotype coverage according to Claes et al. [38]. Effects due to serotype replacement were not included. In contrast to the childhood PCV vaccination, adults receive just one dose of vaccine which is expected to be insufficient to induce any herd effects. In addition, PPV23 is unable to elicit immune memory, so that also a second dose of vaccine would not boost antibody level and therefore would not cause significant herd effects [39]. Hence, herd effects due to adult vaccination were not included in the model.

**Table 4:** Factor for correction of indirect (herd) effects

**Age group**		**IPD**	**CAP**
15 - 39		37,4%*	23.6%*
40 - 44		39.7%*	23.6%*
45 - 49		39.7%*	16,2%*
50 - 64		17.5%*	16,2%*
65+		37.9%*	14.3%*

*Adjusted according to Claes et al. for German serotype coverage based on IPD data from Pilishvili et al. and CAP data from Ray et al. [34].

### Economic parameters

Life years gained (LYG) were the primary health-economic endpoint in our analysis. Prices and utilization of health services per unit were modeled separately. We evaluated the resource use from the perspective of the SHI in Germany, taking into consideration patient co-payments as well as discounts for medications given by the manufacturer and pharmacies as required by legal obligations in Germany. In addition, we estimated costs from a social perspective, including costs for work loss and patients co-payments. To evaluate the costs from the perspective of the SHI, the current German guidelines for the valuation of resource usage were applied. Costs referred to the year 2010.

#### Costs of medical care of pneumococcal associated infections

In the German health care system, inpatient treatment is reimbursed by G-DRG and outpatient treatment by official German Uniform Valuation Scheme (EBM). Table 5 summarizes the costs, which were integrated into the model. We assumed that all IPD cases were treated in hospitals due to the severity of invasive infection. Further we assumed that 20% of all inpatient CAP cases took a more severe course. No cost for long-term disabilities from IPD and CAP were included.

**Table 5:** Direct and indirect cost (Price year = 2010)

	**Age groups**	**Cost per case (incl. co-payments)**	**Source**
**Medical cost**			
PCV13 including administration	All	71.57 € (71.57 €)	Lauer-Taxe (2010), German vaccination agreements of the SHI
PPV23 including administration	All	35.89 € (35.89 €)	Lauer-Taxe (2010), German vaccination agreements of the SHI
IPD	All	9,006.43 € (9,151.90 €)	G-DRG (2010) 87% mean G-DRG T60A-C + T60E + 13% weighted mean G-DRG A06A-B, A07A-E, A09A-F, A11A-G, A13A-G
IPD letal	All	1,445.41 € (1,445.41 €)	G-DRG (2010) T60F
Inpatient CAP, severe	All	8253,93 € (8371,93 €)	G-DRG (2010) E77A
Inpatient CAP, moderate	All	5119,33 € (5185,99 €)	G-DRG (2010) 89% weighted mean G-DRG E77A-D + 11% weighted mean G-DRG A06A-B, A07A-E, A09A-F, A11A-G, A13A-G
Outpatient CAP	5-59	54,75 € (54,75 €)	EBM-Code (2010) 03110, IMS Health Deutschland
Outpatient CAP	60+	59,65 € (59,65 €)	EBM-Code (2010) 03111, IMS Health Deutschland
			
**Non-medical cost**			
Cost for work disability per day	All	95.72 €	Federal Statistical Office 2010

Inpatient costs were derived from official G-DRGs codes (German Diagnosis Related Groups) in 2010 which are calculated based on resource use in 2008 (see Table 5). [40] These costs are lump sums and cover all cost per case which includes, in general, all expenses of the hospital (incl. medication costs). The G-DRG system separately covers the specific need for mechanical ventilation. According to this we assumed 13% [41] and 11% [42] cases with mechanical ventilation for IPD and moderate inpatient CAP respectively. For severe inpatient CAP the DRG code E77A covers cases with a high degree on co-morbidities and complex intensive care treatment.

Regarding the outpatient setting, we applied the official German Uniform Valuation Scheme (EBM). [43] For general outpatient physician visits of SHI-insured persons the doctors are reimbursed via capitation fees per quarter, independent from the number of visits of a patient per quarter. The costs of outpatient care consisted of a quarterly capitation fee in 2010: 6y–59y 30.84 € (EBM 03111: 880*0.035048) > 60 y 35.74 € (EBM 03112: 1020*0.035048) plus an average compensation for prescriptions of 23.91 € per episode generated from the IMS prescription panel [24,43]. The average compensation was calculated on the basis of prescription costs and prescription quantity of ICD10 diagnosis (J12–J18). No extra costs for treating adverse events from vaccination were considered in the model.

#### Costs of the vaccination program

Based on official data and a package size of ten [44] the pharmacy retail price of PCV13 was determined to be 64.62 € per dose PCV13 and 28.94 € per dose for PPV23. There is no official rule for vaccine pricing in Germany. Therefore no manufacturer and pharmacy discounts were subtracted from the pharmacy retail prices for vaccines. Based on a sample of German vaccination agreements between the Association of SHI Physicians and the SHI we calculated an average reimbursement fee of 6.95 € for each injection. It was assumed that the vaccine is administered at the same time as other vaccines so no further visit costs are incurred.

### Indirect costs

Regarding the social perspective cost of work loss and patient’s co-payments were included in the analysis. The cost of work loss was calculated using the human capital approach as proposed by the “Hannoveraner Konsens” [45]. Work loss was expected by an average of 7.4–18.7 days. [46] Assuming an age specific labor participation of ∅ 81.8% for men and ∅ 69.6% for women, daily cost for work disability of 95.72 € were considered as indirect cost in the model [46,47].

### Sensitivity analysis

To access the impact of various parameters on model outcomes and to confirm the robustness of the model, all parameter values were varied individually in one-way sensitivity analysis (SA) and simultaneously in a probabilistic SA with 10.000 iterations. For probabilistic SA, beta distributions were assumed based on published 95% confidence intervals. If confidence intervals or ranges were not reported in the studies, we assumed an upper and lower limit of ±25% of the base case value. To account for skewness in cost data we used gamma distributions. Means and standard errors were derived from the corresponding databases (DRG Browser [40], IMS prescription panel [24]). Means, standard errors, and distributions for all parameters examined in the probabilistic sensitivity analysis are shown in the Appendix.

## Results

Estimated effects on IPD and CAP according to the different vaccination strategies are shown in Table 6. Maintaining vaccination with PPV23, 916,233 episodes and 38,967 deaths related to pneumococcus-induced diseases could be expected each year, which is a reduction of 564 episodes and 97 deaths in comparison to ‘no vaccination’ against pneumococcal diseases. Assuming the PCV7 effectiveness data, with PCV13 vaccination (vaccination in adults older than 50 years) 19,009 episodes and 2,661 deaths could be avoided in comparison to ‘no vaccination’.

**Table 6:** Cases, avoidable episodes and deaths

	**IPD**	**Inpatient CAP**	**Outpatient CAP**	**total**
**Cases without vaccination**				
Episodes	16,145	207,007	693,645	916,797
Deaths	2,730	34,385	1,949	39,064
**Cases with PPV23**				
Episodes	15,581	207,007	693,645	916,233
Deaths	2,633	34,385	1,949	38,967
**Cases with PCV13**				
Episodes	14,784	194,900	688,104	897,788
Deaths	2,478	32,003	1,922	36,403

The results of the base case analysis (Table 7) indicated that switching to PCV13 in adults older than 50 years was cost-effective compared to PPV23 as well as to ‘no vaccination’ and dominated both strategies. According to our model, 22,942 additional life years can be gained by offering PCV13 instead of PPV23 and 24,480 life years can be saved by the PCV13 vaccination program compared to the ‘no vaccination’ scenario. The estimated benefit-cost ratio of PCV13 was 2.09 (2.16 including indirect costs) compared to PPV23 and 1.27 (1.32 including indirect costs) compared to ‘no vaccination’, respectively. Hence, additional cost savings via avoided health care services overcompensated costs related to PCV13 vaccination in adults aged >50 y. The main driver is the prevention of inpatient CAP cases accounting for 80 to 90% of the overall monetary savings of PCV13 over PPV23 and ‘no vaccination’, respectively.

**Table 7:** Cost-effectiveness and benefit-cost ratio

	**PCV13 vs. PPV23**	**PCV13 vs.‘no vaccination’**	**PPV23 vs.‘no vaccination’**
** *Cost-effectiveness* **			
Additional vaccination cost (€)	36,322,624	63,341,927	27,019,302
Cost savings (€)	75,907,677	80,248,574	4,340,897
Life years gained	22,942	24,480	1,537
**ICER (cost per life year gained)**	**−1,725**	**−691**	**14,751**
** *Benefit-cost ratio* **			
**Direct cost**	**2.09**	**1.27**	**0.16**
**Direct cost + indirect cost**	**2.16**	**1.32**	**0.19**

**Figure 2:**
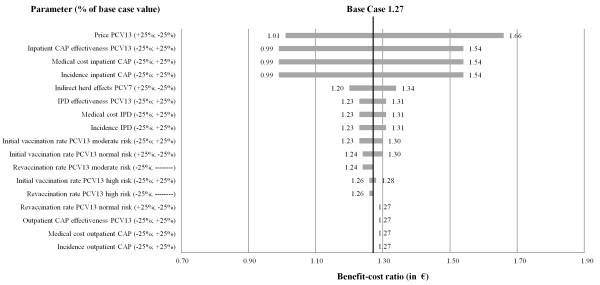
Results of one way sensitivity analyses.

Figure 2 shows results of the one way SA when the same variation factor (+/−25%) was assumed. Overall, the higher the PCV13 price as well as the indirect herd effects, the lower the benefit-cost ratios. Otherwise the higher the incidence rates, the PCV13 effectiveness as well as the medical costs the higher the benefit-cost ratio. Results of one way SA were highly sensitive to variations of the PCV13 price as well as all parameters of inpatient CAP and less sensitive to variations of vaccination rates, indirect herd effects as well as all parameters related to IPD. Variations of parameters related to outpatient CAP had no impact. Lower PCV13 vaccination rates in the normal risk group increased the benefit-cost ratio.

In addition, we tested extreme scenarios, e. g. revaccination frequency of PCV13 = 5 years, no indirect herd-effects through PCV7 vaccination in children, PCV13 completely ineffective in preventing CAP (see Table 8). Even if the monetary net benefit of the PCV13 strategy compared to PPV23 vaccination was negative in some of these scenarios, PCV13 vaccination would be a cost-effective option according to a ICER threshold (assumed between 20,000 and 50,000 €/LYG). Regarding the Maruyama et al. [29] scenario (PPV23 inpatient CAP efficacy = 45%) the PCV13 vaccination program would still be cost-effective with an ICER of 590 €/LYG compared to PPV23, but wouldn’t be a dominant strategy anymore. In a case that PCV13 is completely ineffective in preventing CAP, the ICER of PCV13 compared to PPV23 was 17,983 €/LYG and compared to ‘no vaccination’ 16,440 €/LYG.

**Table 8:** Further one way and multi way sensitivity analyses

	**Benefit-cost ratio PCV13 vs. ‘PPV23**		**Benefit-cost ratio PCV13 vs. ‘no vaccination’**	
	**Only direct cost**	**Including indirect cost**	**Only direct cost**	**Including indirect cost**
**Base case**	2.09 €	2.16 €	1.27 €	1.32 €
**PCV13 revaccination according to current PPV23 adult vaccination recommendations in Germany[**[15]**]**	2.97 €	3.11 €	1.08 €	1.14 €
**Revaccination PCV13 not needed**	16.74 €	17.26 €	2.81 €	2.92 €
**Revaccination PCV13 every 5 years**	0.97 €	1.00 €	0.75 €	0.78 €
**Incidence for in- and outpatient CAP according to Schnoor et al.: -55%[**[48]**]**	1.01 €	1.05 €	0.65 €	0.68 €
**PPV23 effectiveness against IPD upper limit[**[9]**]**	2.07 €	2.14 €	1.27 €	1.32 €
**PPV23 effectiveness against IPD lower limit[**[9]**]**	2.12 €	2.19 €	1.27 €	1.32 €
**PPV23 effective in preventing inpatient CAP according to Maruyama et al. [**[29]**]: 45%**	0.86 €	0.88 €	1.28 €	1.34 €
**PCV13 ineffective in preventing in- and outpatient CAP**	0.17 €	0.17 €	0.16 €	0.18 €
**No indirect herd effects of PCV7 vaccination**	2.51 €	2.60 €	1.54 €	1.61 €

**Figure 3:**
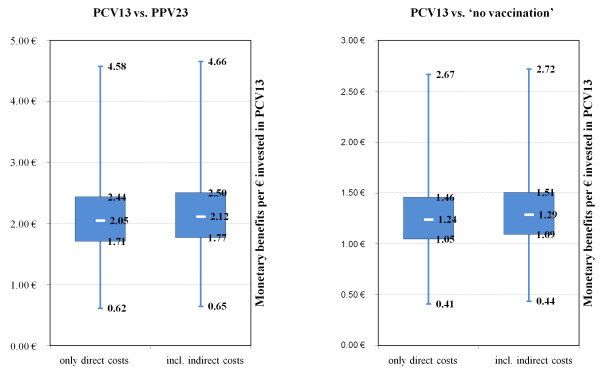
Probabilistic sensitivity analysis boxplots.

**Figure 4:**
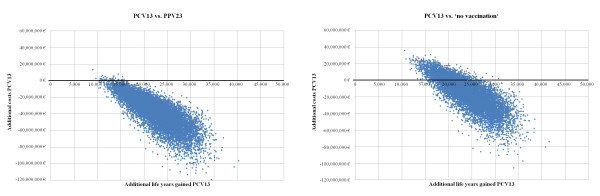
Probabilistic sensitivity analysis scatterplots.

Figure 3 and 4 illustrate the results of the probabilistic sensitivity analysis. The minimum benefit-cost ratios of PCV13 in comparison to PPV23 as well as ‘no vaccination’ were 0.62 (0.65 including indirect cost) and 0.41 (0.44 including indirect cost), respectively. PCV13 dominated PPV23 with a probability of 99.51% and ‘no vaccination’ with a probability of 80.46% according to the simulation.

## Discussion

This study is the first to describe the benefit-cost ratio and cost-effectiveness of a PCV13 vaccination scheme for adults in Germany. The model found that switching from PPV23 to PCV13 in adults older than 50 years was a dominant strategy. Compared to PPV23 as well as ‘no vaccination’, the PCV13 vaccination program revealed the potential to avoid a greater number of yearly cases and deaths due to the potential superior efficacy of PCV13 in IPD and CAP in Germany. One way sensitivity analyses illustrated PCV13 attained a positive net benefit over PPV23 and over ‘no vaccination’ in most of the tested scenarios and an acceptable ICER in all tested scenarios. Variations in input parameters related to inpatient CAP as well as the price of PCV13 had the most significant impact on the results.

The study was mainly limited by lacking clinical data on conjugate vaccine in adults. Except for a study in HIV-infected adults in Malawia using PCV7, no data are available for an effect of conjugate vaccine in adults. Therefore, data on the vaccine efficacy and effectiveness in infants were used as basis for the assumptions and the fact, that immunogenicity data in adults were comparable to immunogenicity data in children. Initial studies for antibody detection underline these assumptions for PCV7 and PCV13 respectively.

There was a lack of evidence for the duration of protection of PPV23 [9]. Different values were published regarding the PPV23 serotype effectiveness against IPD. Therefore, we decided for the base case scenario to use the recommendations according to the Cochrane meta-analysis [9].

In terms of pneumonia four randomized, placebo-controlled trials of vaccination with PPV23 in COPD (chronic obstructive pulmonary disease) have failed to show a significant reduction in mortality, hospitalization, or pneumonia. However, a recent randomized placebo controlled trial in Japanese nursing home residents (85 +/− 8y) revealed a reduction of all cause pneumonia by 45% and of pneumococcal pneumonia by 64%. The reason for these discrepancies remains unclear.

Since data regarding the waning efficacy of PCV13 vaccine are not available, several publications for PPV23, which value the issue of waning, were considered. For PPV, antibody levels to several serotypes decline to pre-vaccination values within 5–10 years corresponding to a decline of protection. Existing immunogenicity data suggest that PCV will provide a long-lasting immunologic memory and protection. [49,50] This issue will be addressed in upcoming publications.

Although evidence of indirect (herd) protection for vaccination in children is demonstrated for North Rhine Westphalia, Saxony and Bavaria we decided to use data from U.S, were evidence is confirmed for the whole population. [51] A study by Ardanuy et al. showed evidence of a decrease in IPD due to PCV7 serotypes for hospitalised adults in Spain for adults aged 18–64 (results not significant) and adults over 65 (significant results). [52] As mentioned in section two, we modelled the cost-effectiveness only for adults (≥18 y). To avoid double counting indirect (herd) effects from children vaccination were excluded. Therefore results in terms of case reduction may be underestimated.

Our results were based on the premise that no replacement of serotypes (i.e., an increase in carriage of and disease from serotypes not included in the vaccine) will take place in Germany, though other studies have noted the importance of collecting data on the impact of serotype evolvement on future evaluations of vaccines for pneumococcal disease. This assumption is obviously in contrast to numerous studies showing serotype shifting after introduction if the vaccine and first signs of serotype shifting have already been observed in Germany. [53] However, since the most of the replacement serotypes, particularly the main replacement serotype 19A, is contained in the novel PCV13, a substantial replacement to this vaccine cannot be foreseen. Some authors also assume that replacement serotypes are less fit and virulent compared to the predominant vaccine serotypes before introduction of PCV 7.

In our base case analysis the ICER of PPV23 compared to ‘no vaccination’ was 14,751 €/LYG, which was comparable to results of a published health economic review. [54] This review showed that PPV23 vaccination was a cost-effective option in vaccination over 65 years old mainly in prevention of IPD in most of the studies. Compared to PCV13, vaccination with PPV23 was an inferior strategy in our model. That goes in line with a recently published study, evaluated the economic impact of using PCV13 in lieu of PPV23 in all adults aged ≥50 y in the US. [55] A published model from the Netherlands estimated the cost-effectiveness of PCV13 compared to ‘no vaccination’ in adults’ aged ≥65 y. The results of the base case analysis indicated that PCV13 was cost-effective but not dominant. However, the model of Rozenbaum et al. had only a time horizon of five years. [56] We simulated a similar scenario in our model by reducing the revaccination frequency of PCV13 from 10 to 5 years. PCV13 would lose its dominance but still remained cost-effective compared to PPV23 as well as ‘no vaccination’.

Most of the previous mentioned health economic studies used the target population, vaccinated routinely in many countries. Effectiveness of PPV23 is discussed controversially. There is a homogeneous consensus that PPV23 has no protection against non-bacteremic pneumonia [9]. Only protection against IPD is assessed differently. Therefore we decided to us no decline in protection for both vaccines even if most of the PPV23 studies considered a decline in protection with different rates.

The focus of this study was to address the benefit of pneumococcal conjugate vaccination in adults. Adverse events are not a problem with conjugate vaccines. For PPV23 only a small impact of adverse events on ICER was shown. Therefore we decided not to implement cost for adverse events in the current model.

Considering the substantial morbidity, mortality and costs associated with pneumococcal disease as well as the limitations of Markov models to simulate the dynamics of transmissible disease, there is a need for more accurate models as soon as valid effectiveness data of PCV13 is published to confirm the cost-effectiveness of this vaccine.

## Conclusions

In conclusion, our analysis indicates that adult PCV13 vaccination in Germany will reduce the burden of pneumococcal disease with substantial health and economic benefits when compared to the currently recommended PPV23 as well as ‘no vaccination’. However, final cost-effectiveness will depend mainly on efficacy data of PCV13 confirmed by clinical trials, particularly in inpatient CAP. Defining the risk population, vaccination rates, indirect (herd) effects and serotype replacement needs further research.

## Appendix A

Sensitivity analysis

The tables below provide information on the parameters included in the probabilistic sensitivity analysis as well as additional results of the one-way and multi-way sensitivity analyses. The means, standard errors and the type of distributions of all parameters examined in the probabilistic analysis are shown in Additional file 1: Table S1. Additional file 2: Table S2 illustrates the results of all one-way and multi-way sensitivity analyses conducted.

## Competing interest

The paper was supported by Pfizer Deutschland GmbH. Mathias Pletz is a member of the Pfizer advisory board and has received research grants of Sanofi Pasteur MSD.

## Authors’ contributions

AK constructed and implemented the model in Excel, performed the analysis of the results and drafted the manuscript. UT was responsible for the literature review, data acquisition and drafted the manuscript. MP helped to draft the manuscript and revised it critically for important intellectual content. JMGvdS reviewed the manuscript and revised it critically for important intellectual content. All authors read and approved the final manuscript.

## Supplementary Material

Additional file 1 **Table S1.**Means, standard errors and distributions of parameters examined in the probabilistic sensitivity analysis. Click here for file

Additional file 2 **Table S2.** One-way and multi-way sensitivity analyses. Click here for file
