# Supplementary material for: Potential cost-effectiveness and benefit-cost ratios of adult pneumococcal vaccination in Germany
Source: Health Econ Rev. 2012 Mar 30;2:4. doi: 10.1186/2191-1991-2-4 (PMC3463422; doi:10.1186/2191-1991-2-4)
Supplement: Additional file 1 — Table S1.Means, standard errors and distributions of parameters examined in the probabilistic sensitivity analysis. [file 2191-1991-2-4-S1.doc]

**Table S1.**  Means, standard errors and distributions of parameters examined in the probabilistic sensitivity analysis

|  | **Mean** | **Standard error** | **Distribution** |
| --- | --- | --- | --- |
| **IPD incidence per 100,000 individuals** | | | |
| 18 - 39 | 0.500 | 0.041 | beta |
| 40 - 49 | 6.800 | 0.567 | beta |
| 50 - 59 | 13.300 | 1.108 | beta |
| 60 - 69 | 23.000 | 1.917 | beta |
| 70 - 79 | 41.600 | 3.467 | beta |
| 80+ | 53.700 | 4.475 | beta |
|  |  |  |  |
| **inpatient CAP incidence per 100,000 individuals** | | | |
| 18 - 39 | 48.900 | 4.075 | beta |
| 40 - 49 | 80.400 | 6.700 | beta |
| 50 - 59 | 149.700 | 12.475 | beta |
| 60 - 69 | 350.400 | 29.200 | beta |
| 70 - 79 | 768.700 | 64.058 | beta |
| 80+ | 1,897.800 | 158.150 | beta |
|  |  |  |  |
| **outpatient CAP incidence per 100,000 individuals** | | | |
| 18 - 39 | 574.700 | 47.892 | beta |
| 40 - 49 | 777.300 | 64.775 | beta |
| 50 - 59 | 1,074.000 | 89.500 | beta |
| 60 - 69 | 1,459.300 | 121.608 | beta |
| 70 - 79 | 1,465.800 | 122.150 | beta |
| 80+ | 2,025.200 | 168.767 | beta |
|  |  |  |  |
| **IPD case fatality rates** | | | |
| 18 - 39 | 14.900 % | 1.242 % | beta |
| 40 - 49 | 14.900 % | 1.242 % | beta |
| 50 - 59 | 14.900 % | 1.242 % | beta |
| 60 - 69 | 14.900 % | 1.242 % | beta |
| 70 - 79 | 16.500 % | 1.375 % | beta |
| 80+ | 27.400 % | 2.283 % | beta |
|  |  |  |  |
| **inpatient CAP case fatality rates** | | | |
| 18 - 39 | 1.600 % | 0.133 % | beta |
| 40 - 49 | 4.300 % | 0.358 % | beta |
| 50 - 59 | 6.300 % | 0.525 % | beta |
| 60 - 69 | 10.800 % | 0.900 % | beta |
| 70 - 79 | 16.800 % | 1.400 % | beta |
| 80+ | 22.600 % | 1.833 % | beta |
|  |  |  |  |
| **outpatient CAP case fatality rates** | | | |
| 18 - 39 | 0.000 % |  |  |
| 40 - 49 | 0.000 % |  |  |
| 50 - 59 | 0.500 % | 0.042 % | beta |
| 60 - 69 | 0.500 % | 0.042 % | beta |
| 70 - 79 | 0.500 % | 0.042 % | beta |
| 80+ | 0.500 % | 0.042 % | beta |
|  |  |  |  |
| **PCV13 IPD serotype coverage** | | | |
| 18 - 39 | 67.900 % | 5.568 % | beta |
| 40 - 49 | 75.400 % | 6.283 % | beta |
| 50 - 59 | 63.400 % | 5.283 % | beta |
| 60 - 69 | 63.900 % | 5.325 % | beta |
| 70 - 79 | 71.400 % | 5.950 % | beta |
| 80 - 89 | 74.800 % | 6.233 % | beta |
| 90+ | 76.200 % | 6.350 % | beta |
|  |  |  |  |
| **PCV13 IPD serotype coverage** | | | |
| 18 - 39 | 83,300 % | 6.254 % | beta |
| 40 - 49 | 78,500 % | 6.542 % | beta |
| 50 - 59 | 78,900 % | 6.575 % | beta |
| 60 - 69 | 83,600 % | 6.217 % | beta |
| 70 - 79 | 78,300 % | 6.525 % | beta |
| 80 - 89 | 84,500 % | 6.104 % | beta |
| 90+ | 90,500 % | 5.354 % | beta |
|  |  |  |  |
| **PCV13 IPD efficacy** | | | |
| 18+ | 93.900 % | 3.150 % | beta |
|  |  |  |  |
| **PPV23 IPD efficacy** | | | |
| 18+ | 74.000 % | 4.833 % | beta |
|  |  |  |  |
| **PCV13 inpatient CAP effectiveness** | | | |
| 18+ | 25.500 % | 5.700 % | beta |
|  |  |  |  |
| **PPV23 inpatient CAP effectiveness** | | | |
| 18+ | 0.000 % | 0.000 % |  |
|  |  |  |  |
| **PCV13 inpatient CAP effectiveness** | | | |
| 18+ | 6.000 % | 2.083 % | beta |
|  |  |  |  |
| **PPV23 inpatient CAP effectiveness** | | | |
| 18+ | 0.000 % | 0.000 % |  |
|  |  |  |  |
| **PCV7 IPD indirect herd effects** | | | |
| 18 - 39 | 37.372 % | 3.114 % | beta |
| 40 - 49 | 39.720 % | 3.310 % | beta |
| 50 - 64 | 17.465 % | 1.455 % | beta |
| 65+ | 37.896 % | 3.158 % | beta |
|  |  |  |  |
| **PCV7 CAP indirect herd effects** | | | |
| 18 - 44 | 23.569 % | 1.964 % | beta |
| 45 - 64 | 16.151 % | 1.346 % | beta |
| 65+ | 14.273 % | 1.189 % | beta |
|  |  |  |  |
| **PCV13 price including administration** | | | |
|  | 71.570 € | 2.460 € | gamma |
|  |  |  |  |
| **PPV23 price including administration** | | | |
|  | 35.890 € | 2.460 € | gamma |
|  |  |  |  |
| **Inpatient costs G-DRG** | | | |
| B72A | 4,985.510 € | 3,057.720 € | gamma |
| B72B | 2,631.500 € | 1,182.010 € | gamma |
| T01A | 12,314.860 € | 5,612.010 € | gamma |
| T01B | 8,257.040 € | 3,681.500 € | gamma |
| T60A | 8,396.240 € | 4,239.510 € | gamma |
| T60B | 5,988.140 € | 2,647.490 € | gamma |
| T60C | 5,053.640 € | 2,279.750 € | gamma |
| T60D | 3,573.080 € | 1,329.420 € | gamma |
| T60E | 2,946.060 € | 1,178.750 € | gamma |
| T60F | 1,429.410 € | 1,020.450 € | gamma |
| E77A | 8,253.930 € | 3,656.920 € | gamma |
| E77B | 5,412.260 € | 2,309.960 € | gamma |
| E77C | 4,162.110 € | 2,029.910 € | gamma |
| E77F | 2,859.890 € | 187.860 € | gamma |
| E77G | 2,244.860 € | 861.640 € | gamma |
| D62Z | 1,217.090 € | 493.210 € | gamma |
| D63Z | 1,292.200 € | 416.540 € | gamma |
| D06A | 3,899.590 € | 1,348.870 € | gamma |
| D06B | 2,758.680 € | 851.200 € | gamma |
| D06C | 2,493.230 € | 651.090 € | gamma |
| D01B | 29,601.340 € | 2,585.540 € | gamma |
| A06A | 205,208.445 € | 44,538.240 € | gamma |
| A06B | 139,374.905 € | 32,652.950 € | gamma |
| A07A | 118,357.935 € | 24,086.720 € | gamma |
| A07B | 99,180.025 € | 19,749.840 € | gamma |
| A07C | 89,305.595 € | 19,670.880 € | gamma |
| A07D | 78,535.675 € | 17,575.120 € | gamma |
| A07E | 65,232.540 € | 17,062.240 € | gamma |
| A09A | 76,696.615 € | 19,501.810 € | gamma |
| A09B | 71,506.980 € | 16,731.860 € | gamma |
| A09C | 57,212.195 € | 14,486.150 € | gamma |
| A09D | 52,160.790 € | 12,186.120 € | gamma |
| A09E | 43,975.170 € | 9,890.290 € | gamma |
| A09F | 35,717.430 € | 6,973.360 € | gamma |
| A11A | 54,946.425 € | 15,812.630 € | gamma |
| A11B | 40,185.865 € | 11,095.370 € | gamma |
| A11C | 37,385.205 € | 10,843.940 € | gamma |
| A11D | 33,941.475 € | 7,808.690 € | gamma |
| A11E | 31,420.280 € | 8,099.460 € | gamma |
| A11F | 26,293.750 € | 7,001.180 € | gamma |
| A11G | 21,392.595 € | 5,680.810 € | gamma |
| A13A | 37,781.865 € | 9,614.750 € | gamma |
| A13B | 29,226.630 € | 7,924.660 € | gamma |
| A13C | 25,812.950 € | 7,330.430 € | gamma |
| A13D | 22,113.795 € | 6,366.190 € | gamma |
| A13E | 20,614.300 € | 6,045.560 € | gamma |
| A13F | 13,901.130 € | 4,671.260 € | gamma |
| A13G | 11,172.590 € | 3,532.790 € | gamma |
|  |  |  |  |
| **Outpatient CAP costs** | | | |
| 18 - 59 | 54.750 € | 8.213 € | gamma |
| 60+ | 59.650 € | 8.948 € | gamma |
|  |  |  |  |
| **Cost for work disability per day** | | | |
|  | 95.720 € | 14.358 € | gamma |
