# Supplementary material for: Potential cost-effectiveness and benefit-cost ratios of adult pneumococcal vaccination in Germany
Source: Health Econ Rev. 2012 Mar 30;2:4. doi: 10.1186/2191-1991-2-4 (PMC3463422; doi:10.1186/2191-1991-2-4)
Supplement: Additional file 2 — Table S2. One-way and multi-way sensitivity analyses. [file 2191-1991-2-4-S2.doc]

**Table S2**  One-way and multi-way sensitivity analyses

|  |  | **Benefit-cost ratio PCV13 vs. ‘PPV23** | | **Benefit-cost ratio PCV13 vs. ‘no vaccination’** | |
| --- | --- | --- | --- | --- | --- |
|  | | Only direct cost | Including indirect cost | Only direct cost | Including indirect cost |
| **Base case** | | 2.09 € | 2.16 € | 1.27 € | 1.32 € |
| **Initial vaccination rate PCV13 (all risk groups): -25 %** | | 2.50 € | 2.59 € | 1.25 € | 1.31 € |
| **Initial vaccination rate PCV13 (all risk groups): +25 %** | | 1.89 € | 1.96 € | 1.27 € | 1.32 € |
| **Revaccination PCV13 not needed** | | 16.74 € | 17.26 € | 2.81 € | 2.92 € |
| **Revaccination PCV13 every 5 years** | | 0.97 € | 1.00 € | 0.75 € | 0.78 € |
| **Incidence for all diseases: -25 %** | | 1.57 € | 1.62 € | 0.95 € | 0.99 € |
| **Incidence for all diseases: +25 %** | | 2.61 € | 2.70 € | 1.58 € | 1.65 € |
| **Incidence for in- and outpatient CAP according to Schnoor et al.: -55 % [48]** | | 1.01 € | 1.05 € | 0.65 € | 0.68 € |
| **Mortality rates for all diseases: -25 %** | | 2.10 € | 2.17 € | 1.27 € | 1.33 € |
| **Mortality rates for all diseases: +25 %** | | 2.08 € | 2.15 € | 1.26 € | 1.31 € |
| **Effectiveness PCV 13 for all diseases: -25 %** | | 1.54 € | 1.59 € | 0.95 € | 0.99 € |
| **Effectiveness PCV 13 for all diseases: +25 %** | | 2.59 € | 2.68 € | 1.55 € | 1.61 € |
| **PCV13 ineffective in preventing inpatient CAP** | | 0.17 € | 0.20 € | 0.17 € | 0.20 € |
| **PPV23 effective in preventing inpatient CAP according to Maruyama et al. [29]:** **45 %** | | 0.86 € | 0.88 € | 1.28 € | 1.34 € |
| **PCV13 ineffective in preventing outpatient CAP** | | 2.08 € | 2.13 € | 1.26 € | 1.30 € |
| **PCV13 ineffective in preventing in- and outpatient CAP** | | 0.17 € | 0.17 € | 0.16 € | 0.18 € |
| **No indirect herd effects of PCV7 vaccination** | | 2.51 € | 2.60 € | 1.54 € | 1.61 € |
| **All medical cost and cost for work disability per day: -25 %** | | 1.57 € | 1.62 € | 0.95 € | 0.99 € |
| **All medical cost and cost for work disability per day: +25 %** | | 2.61 € | 2.70 € | 1.58 € | 1.65 € |
